# rmcorrShiny: A web and standalone application for repeated measures correlation

**DOI:** 10.12688/f1000research.55027.1

**Published:** 2021-07-30

**Authors:** Laura R. Marusich, Jonathan Z. Bakdash

**Affiliations:** 1 U.S. Army Combat Capabilities Development Command Army Research Laboratory South at the University of Texas at Arlington, Arlington, TX, 76019, USA; 2 U.S. Army Combat Capabilities Development Command Army Research Laboratory South at the University of Texas at Dallas, Richardson, TX, 75080, USA; 3 Department of Psychology and Special Education, Texas A&M University-Commerce, Commerce, TX, 75428, USA

**Keywords:** repeated measures correlation, statistics, multilevel modeling, Shiny, correlation, regression, repeated measures, within-participants

## Abstract

We describe a web and standalone Shiny app for calculating the common, linear within-individual association for repeated assessments of paired measures with multiple individuals: repeated measures correlation (rmcorr). This tool makes rmcorr more widely accessible, providing a graphical interface for performing and visualizing the output of analysis with rmcorr. In contrast to rmcorr, most widely used correlation techniques assume paired data are independent. Incorrectly analyzing repeated measures data as independent will likely produce misleading results. Using aggregation or separate models to address the issue of independence may obscure meaningful patterns and will also tend to reduce statistical power. rmcorrShiny (repeated measures correlation Shiny) provides a simple and accessible solution for computing the repeated measures correlation. It is available at: https://lmarusich.shinyapps.io/shiny_rmcorr/.

## Introduction

The most common techniques for calculating the correlation between two variables ( *e.g.,* the Pearson correlation coefficient) assume that each pair of data points arises from an independent observation. Take, for example, a study that calculates the correlation between age and the volume of a specific brain region for a sample of people. In this example, each individual contributes a data point consisting of a brain volume and an age. However, it is not uncommon for studies to use repeated measures designs, such as a study by Raz *et al.* ^1^ that collected the brain region volume and age at two different time points. Each participant in this study contributed two (repeated) data points of paired measures. Repeated measures of the same individual are no longer independent observations and should not be analyzed as such. Erroneously modeling repeated measures data as independent observations is surprisingly prevalent in published research, even though such results will generally be misleading. ^2- 4^ A common way to resolve this problem is to use aggregated data: first taking an average of the repeated measures data of each person so that every individual again contributes a single paired data point, and then calculating the correlation from these averages (between-participants). Another possibility is to use separate models to analyze each paired data point, removing the dependency. For example, the study by Raz *et al.* ^1^ computed separate correlations between brain region volume and age at each of the two time points.

Instead of aggregation or separate models, an alternative solution is to calculate the repeated measures correlation, ^5- 7^ which assesses the common intra-individual (within-participants) association for paired repeated measures data. The repeated measures correlation technique is conceptually similar to a null multilevel model, with a common (fixed effect) slope but varying (random effect) intercept for each individual. Calculating the repeated measures correlation has multiple potential benefits. It is simpler and more straightforward to implement than a multilevel model, with the potential for much greater statistical power than aggregation. In addition, repeated measures correlation can provide insights into patterns within individuals that may be obscured by aggregation or use of separate models. ^5^

We previously developed the rmcorr package ^8^ in R ^9^ to make the repeated measures correlation technique widely available for researchers; it has since also been adapted as a function in the Pingouin statistics package ^10^ for Python. However, the use of both of these packages requires some facility with programming languages, which may limit accessibility.

## Methods

### Implementation

**Figure 1.**
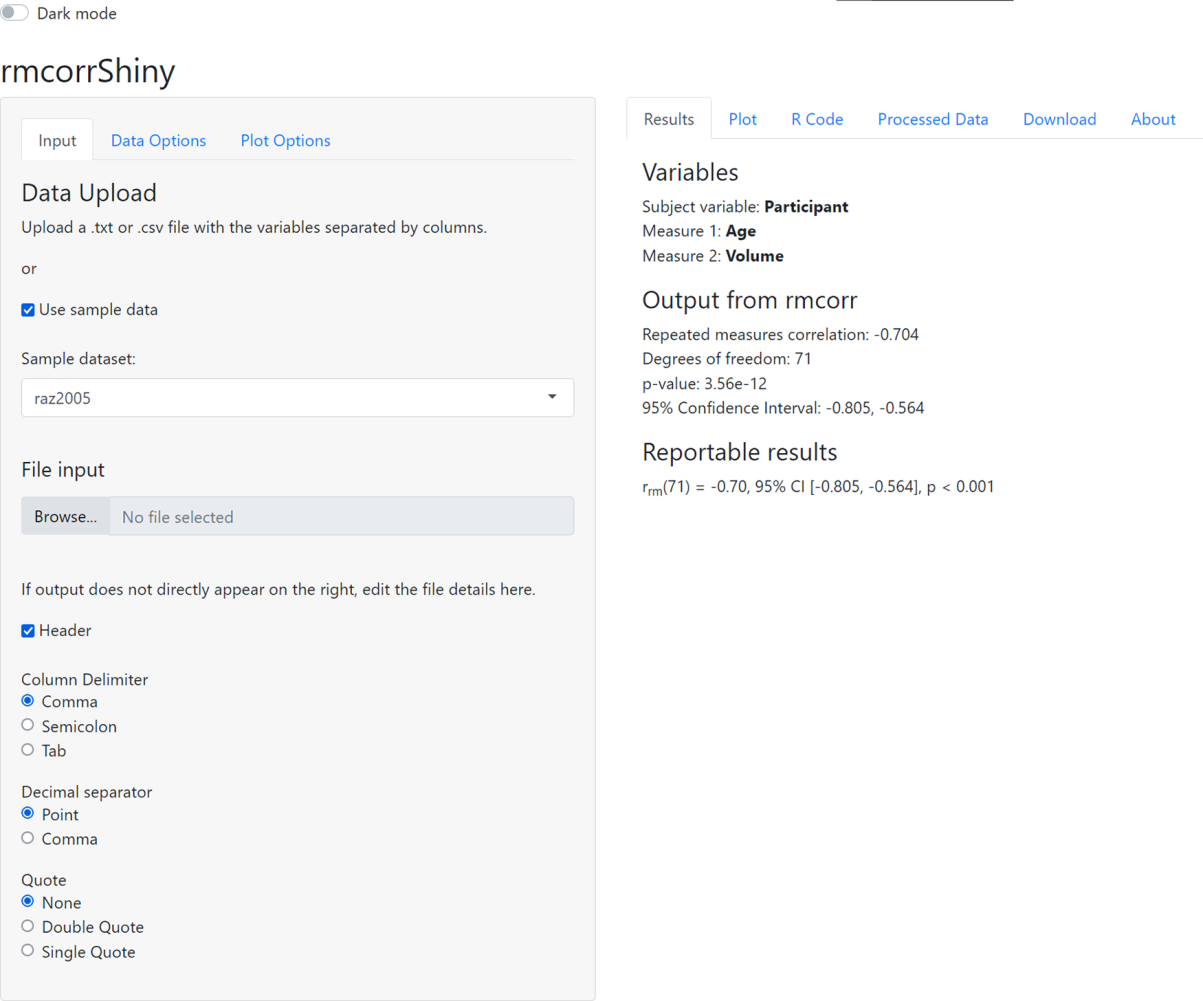
Graphical interface for rmcorrShiny.

Here we introduce rmcorrShiny, a Shiny ^11^ app, which provides an intuitive graphical interface for computing and plotting the repeated measures correlation. An example of the interface is shown in Figure 1.

The interface consists of two panels, corresponding roughly to input (left) and output (right), with each containing multiple subpanels or tabs. The left panel has tabs for **Input**: uploading data or using sample data, **Data Options**: selecting variables for analysis, and **Plot Options**: customizing labels and plot appearance. The right panel has tabs for **Results**: statistical output from rmcorr, **Plot**: corresponding visualization of the rmcorr results, **R code**: the custom R code generating the analysis and plot for the selected data/options, **Processed Data**: summary statistics on data and raw viewer, **Download**: buttons to download the plot and generated R code, and **About**: information about the app.

The primary features of rmcorrShiny include:

• The ability to import data in a variety of different file formats or to use one of four included sample datasets, described below.
• Options for bootstrapping the confidence interval (CI) for the rmcorr effect size.
• The display of raw data and the output from rmcorr as well as formatted output for reporting scientific results.
• Multiple options to generate and customize rmcorr plots, making use of the ggplot2 package ^12, 13^ and palettes from the RColorBrewer ^14^ and pals ^15^ packages.
• Customized R code generated using the data and options chosen by the user that can be directly pasted and executed in R to produce the same output as in rmcorrShiny, or as a starting point for additional customization in R.
• The ability to download plots (in multiple file formats) or a .zip file of all output.

Note that many features in rmcorrShiny, including the panel interface, were based on modifications of code from the Raincloud-shiny app. ^16^

### Operation

rmcorrShiny can be used in a web browser here, or the package can be installed from Github and run locally in R, using the following commands:

devtools::install_github("lmarusich/rmcorrShiny")library(rmcorrShiny)rmcorrShiny::rmcorrShiny()



## Use case

**Figure 2.**
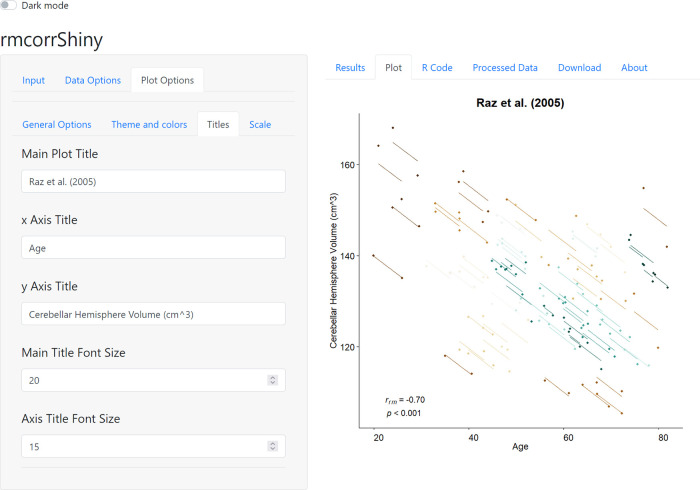
Plot options and rmcorr plot of sample neuroscience dataset *raz2005*. ^1^

As a use case, we use rmcorrShiny to compute and plot the repeated measures correlation of *raz2005* ^1^, one of the four included sample datasets. The right panel of Figure 2 shows the rmcorr plot for this data. Because a variety of patterns in data can produce similar or even identical statistical results for rmcorr, as well as other models, we highly recommend visualization to aid in interpreting results. In Figure 2, the x-axis is age and the y-axis is volume of a brain area, the cerebellar hemisphere. Each participant, plotted in a different color, contributes two paired data points representing two assessments of age and brain volume. Paired age and brain volume were measured twice per participant about five years apart. The corresponding lines depict the repeated measures correlation model. Note the large negative slope and close fit of the lines to many of the data points. This shows a strong pattern of a common decrease in the volume of this brain area over time, across different ages. The lower left corner of the plot shows the effect size for rmcorr and its *p*-value. 

As mentioned previously, the pattern of results may vary, depending on whether the analysis is within- or between-individuals. ^17, 18^ This can be observed in the current example; when the *raz2005* dataset is analyzed using aggregation or separate models (between-individual analyses), the magnitude of the negative association is diminished in comparison to rmcorr (within-individual analysis; see Figure 5 in Bakdash & Marusich ^5^).

## Limitations

rmcorrShiny has the advantage of making the useful and already fairly simple-to-use rmcorr technique even more accessible and easy to implement and interpret. However, its limitations are the same as rmcorr’s: it does not have all of the capabilities of multilevel modeling and thus it is not a replacement. We recommend using multilevel modeling if: there are more than two variables of interest (in addition to participants or subjects), data with more than two levels, simultaneous modeling of within- and between-participants is needed, visual patterns suggest varying slopes by individual and/or non-linear associations, or time needs to be explicitly included in the model ( *i.e.*, modeling change over time as a separate variable; note in Figure 2 age is a measure of time, but it is not a separate variable). For more information about multilevel modeling, see Aarts *et al*. ^2^ and the **About** tab in rmcorrShiny.

## Conclusion

This paper demonstrates the rmcorrShiny app for graphing and analyzing paired, repeated measures data without requiring programming knowledge. By increasing the accessibility of the rmcorr technique, we aim to increase analyses of patterns within-participants as an alternative to other approaches. Such approaches include incorrectly modeling repeated measures data as independent, which is highly problematic, as well as analyses performed only on aggregated data or using separate models. In particular, this app may be useful for visualization and analysis of a common linear pattern across participants and could be a informative precursor complementing multilevel modeling.

## Data availability

The rmcorrShiny app contains sample datasets from four previously published papers.

• *bland1995*: Health science data with repeated measures, by participant, of pH (degree of base or acidity) and PaCO2 (partial pressure of carbon dioxide). ^6^
• *gilden2010*: Psychology data of reaction time and accuracy for a visual search task by participant, in four repeated blocks (both dependent measures are averaged by block). ^19^
• *marusich2016*: Psychology data of dyads working together to capture High Value Targets (lower task time is better performance) and their averaged Mission Awareness Rating Scale (MARS) score for each block, repeated three times. MARS evaluates subjective situation awareness (”knowing what is going on”), higher values indicate better situation awareness. ^20^
• *raz2005*: Neuroscience data containing the volume of a brain area (cerebellar hemisphere) measured twice per participant at two different ages, approximately 5 years apart. ^1^

The csv files for datasets can be directly downloaded from Github ( https://github.com/lmarusich/rmcorrShiny/tree/master/inst/shiny).

## Software availability

• Software available from: https://lmarusich.shinyapps.io/shiny_rmcorr/
• Source code available from: https://github.com/lmarusich/rmcorrShiny
• Archived source code at time of publication: http://doi.org/10.5281/zenodo.5082964 ^21^
• License: GPL-3
