# Supplementary material for: rmcorrShiny: A web and standalone application for repeated measures correlation
Source: F1000Res. 2021 Nov 8;10:697. Originally published 2021 Jul 30. [Version 2] doi: 10.12688/f1000research.55027.2 (PMC8456376; doi:10.12688/f1000research.55027.2)
Supplement: Peer review history

# Peer review: Reviewer response for version 2

**DOI:** 10.5256/f1000research.78432.r99423

**Published:** 2021-11-09

**Authors:** Emmeke Aarts, Sebastian Mildiner Moraga

**Affiliations:** 1 Department of Methodology and Statistics, Utrecht University, Utrecht, The Netherlands; 2 Department of Methodology and Statistics Utrecht, Utrecht University, Utrecht, The Netherlands

No further comments.

Are the conclusions about the tool and its performance adequately supported by the findings presented in the article?

Yes

Is the rationale for developing the new software tool clearly explained?

Yes

Is the description of the software tool technically sound?

Yes

Are sufficient details of the code, methods and analysis (if applicable) provided to allow replication of the software development and its use by others?

Yes

Is sufficient information provided to allow interpretation of the expected output datasets and any results generated using the tool?

Yes

Reviewer Expertise:

Methodology and statistics, multilevel modelling

We confirm that we have read this submission and believe that we have an appropriate level of expertise to confirm that it is of an acceptable scientific standard.

# Peer review: Reviewer response for version 1

**DOI:** 10.5256/f1000research.58566.r93337

**Published:** 2021-09-21

**Authors:** Emmeke Aarts, Sebastian Mildiner Moraga

**Affiliations:** 1 Department of Methodology and Statistics, Utrecht University, Utrecht, The Netherlands; 2 Department of Methodology and Statistics Utrecht, Utrecht University, Utrecht, The Netherlands

The authors present an online (and open source), user-friendly and very complete software tool within the R Shiny framework to infer the linear within-individual correlation for repeated observations within multiple individuals for two outcomes. The R shiny app is based on the previously developed R package rmcorr published on CRAN, making both analyses using rmcorr and data visualization accessible to an audience not comfortable with R. The manuscript provides a concise and clear rationalization for the development of the software tool, explaining why dependency due to repeated observations needs to be accommodated in the used analysis method. We especially enjoyed the clear and aesthetically pleasing graphs making use of ggplot2 provided with the app, and the numerous customization options for the graphs: from more basic matters like specifying the titles of the axes to even allowing the user to specify the color palette. Also, the code provided in the R Code tab is potentially very helpful and insightful to users wanting to become more acquainted with doing these analyses in R or wanting to slightly modify some steps of the analysis or visualization. The fact that multiple sample datasets are available within the tool is also a big plus. In conclusion, the app is a very nice resource valuable to a wide audience. It's a tool we will recommend to applied researchers analyzing dependent data and we look forward to use it for teaching purposes.  

 We have some minor suggestions that might help to further improve the tool and the utility for other researchers:

- It is unclear what happens when the data includes missing observations. Will these cases be ignored using listwise deletion? It would be nice if the authors could provide a small comment on this in the manuscript.
- We can imagine that for some users it might be very useful and insightful if the app includes a tab that provides (visual) information on whether the statistical assumptions of the analysis method are met. It would be a very nice addition to an already very complete app.
- When trying out the app, an error was produced on the max upload size (the app did work when using a different dataset of a smaller size), if there is a restriction on the maximum size for used datasets it would be nice if a mention of this could be included in the manuscript.

Are the conclusions about the tool and its performance adequately supported by the findings presented in the article?

Yes

Is the rationale for developing the new software tool clearly explained?

Yes

Is the description of the software tool technically sound?

Yes

Are sufficient details of the code, methods and analysis (if applicable) provided to allow replication of the software development and its use by others?

Yes

Is sufficient information provided to allow interpretation of the expected output datasets and any results generated using the tool?

Yes

Reviewer Expertise:

Methodology and statistics, multilevel modelling

We confirm that we have read this submission and believe that we have an appropriate level of expertise to confirm that it is of an acceptable scientific standard.

# Response

**Published:** 2021-10-26

**Authors:** Laura Marusich

**Affiliations:** U.S. Army Research Laboratory, USA

- *It is unclear what happens when the data includes missing observations. Will these cases be ignored using listwise deletion? It would be nice if the authors could provide a small comment on this in the manuscript.*
- **Response:  **That is correct, missing observations are ignored using listwise deletion. We added this key detail to the paper. We also discovered and fixed a bug with plotting data that had missing observations.
- *We can imagine that for some users it might be very useful and insightful if the app includes a tab that provides (visual) information on whether the statistical assumptions of the analysis method are met. It would be a very nice addition to an already very complete app.*
- **Response: **This is an excellent idea and desirable feature, but we think it will be very challenging to implement in Shiny. For now, we have added it as a wish-list item. In the paper, we added a sentence that the statistical assumptions for rmcorr are the same as ANCOVA, with the exception of independence, and again emphasized visualization of the data and model. The latter, to some extent, can be used to visually check assumptions such as homogeneity of variance and linearity.
- *When trying out the app, an error was produced on the max upload size (the app did work when using a different dataset of a smaller size), if there is a restriction on the maximum size for used datasets it would be nice if a mention of this could be included in the manuscript.*
- **Response**: Thanks for testing and finding this issue. We modified the web app to increase the maximum file size to 30 MB. Also, we noted this in the paper and added that a file exceeding 30 MB can be loaded by running the app locally.

# Peer review: Reviewer response for version 1

**DOI:** 10.5256/f1000research.58566.r93336

**Published:** 2021-09-10

**Authors:** Ronan McGarrigle

**Affiliations:** 1 Department of Psychology, University of Bradford, Bradford, UK

The authors outline the development of a user-friendly software tool ('rmcorrShiny') for running repeated-measures correlation (rmcorr) analysis without the need for any prior programming knowledge. The paper provides a compelling rationale for the development of this novel tool and all materials are shared in an open and transparent way. I believe this has the potential to be a highly useful resource for researchers across a variety of disciplines. I particularly enjoyed the seamless integration with ggplot2 in R to provide detailed and aesthetically-pleasing visualizations of the rmcorr data, which can be very easily created and modified by the user. I also liked the 'reportable results' section of the app, which I believe novice users will find especially useful. Overall, I believe this is an excellent resource that I look forward to using myself and have no doubt others will follow suit.

 I just have a few minor suggestions that I hope will help to promote the utility of this tool for researchers:

- As mentioned above, I found the shiny app extremely intuitive and straight-forward to use. To test it with some of my own data, I uploaded two data sets. First, I uploaded a dataset from my own previous study for which I had run rmcorr analysis to see if the plots/findings would replicate on the app. I am pleased to report that all of the values provided in the 'results' section on the app were consistent with my own previous analyses, and the plot looked great! To test the app once more, this time I fabricated a dataset in excel (5 repeated measures, two variables, 10 subjects) and tried to plot/analyze the data once again using the app. The 'Results', 'Processed data', and 'R Code' sections all looked fine and appeared similar in format to both the previous analysis and the sample data provided on the app (the columns in the .csv file were correctly identified as variables, etc.). However, the plot tab did not appear to work on this occasion - I received the following error message; 'Error: an error has occurred. Check your logs or contact the app author for clarification'. I'm sure there will be a very simple fix, but I just wanted to flag this in case of a bug. Please feel free contact me if you would like me to share the specific datasets so that you can try to replicate the issue yourselves.
- Introduction, paragraph 2: When describing the benefits of rmcorr in the Introduction, I wonder if it might be worth providing a more concrete example to highlight when this approach may be particularly useful - e.g., as a way to assess changes over time between two adjacent measures. A more concrete example might help to really drive home this point for interested readers. For example, researchers are often interested in examining to what extent changes over time in one measure (e.g., self-report) mirror changes over time in another measure (e.g., physiological). While data aggregation would collapse over (and thus ignore) this kind of association, rmcorr provides a powerful way to assess within-subject change between these paired measures.
- Limitations, line 4: I just had to re-read this sentence a few times. I wonder if changing this to 'if there are more than two *outcome* variables of interest' would help to clarify this point (instead of what is currently in brackets)?
- Limitations, line 4: I wonder if it would be helpful to clarify what you mean by 'data with more than two levels', with an example perhaps? I guess I'm just wondering if data collected at two separate time points constitutes two levels in this context (I'm guessing not, but I think it might just be useful to clarify).
- Conclusions, lines 2-5: I found the first sentence of this paragraph ('we aim to increase analyses of patterns within-participants') a little tricky to parse. As a possible alternative that I hope does not misinterpret the point you are trying to make (if so, please feel free to ignore), how about something like: 'we aim to bolster the researcher’s toolbox by providing a simple but powerful tool for examining associations present at the within-subject level in a way that does not violate independence assumptions or require use of separate models or data aggregation'.
- Conclusions, line 6: 'an' informative precursor.

Are the conclusions about the tool and its performance adequately supported by the findings presented in the article?

Yes

Is the rationale for developing the new software tool clearly explained?

Yes

Is the description of the software tool technically sound?

Yes

Are sufficient details of the code, methods and analysis (if applicable) provided to allow replication of the software development and its use by others?

Yes

Is sufficient information provided to allow interpretation of the expected output datasets and any results generated using the tool?

Yes

Reviewer Expertise:

Experimental Psychology, Cognitive Science

I confirm that I have read this submission and believe that I have an appropriate level of expertise to confirm that it is of an acceptable scientific standard.

# Response

**Published:** 2021-10-26

**Authors:** Laura Marusich

**Affiliations:** U.S. Army Research Laboratory, USA

- *I received the following error message; 'Error: an error has occurred. Check your logs or contact the app author for clarification'. I'm sure there will be a very simple fix, but I just wanted to flag this in case of a bug.*
- **Response:** We contacted Dr. McGarrigle for more information and found the source of the bug. Spaces in variable names were causing errors in plotting. This has been fixed in the current version of the app, thanks.
- *Introduction, paragraph 2: A more concrete example to highlight when this approach may be particularly useful - e.g., as a way to assess changes over time between two adjacent measures. A more concrete example might help to really drive home this point for interested readers.*
- **Response:** We have attempted to draw more connections between the conceptual description of the repeated measures correlation technique and the use case showing analysis of data from Raz et al. (2005), to demonstrate that the within-individual patterns revealed by rmcorr may be different from between-individual patterns.
- *Limitations, line 4*
  - *I wonder if changing this to 'if there are more than two outcome variables of interest' would help to clarify this point (instead of what is currently in brackets)*
  - *I wonder if it would be helpful to clarify what you mean by 'data with more than two levels', with an example perhaps?*
- **Response:**  We agree this sentence is difficult to understand and have rewritten it.
- *Conclusions, lines 2-5: I found the first sentence of this paragraph ('we aim to increase analyses of patterns within-participants') a little tricky to parse. As a possible alternative that I hope does not misinterpret the point you are trying to make (if so, please feel free to ignore), how about something like: 'we aim to bolster the researcher’s toolbox by providing a simple but powerful tool for examining associations present at the within-subject level in a way that does not violate independence assumptions or require use of separate models or data aggregation'.*
- **Response:** Thanks! We switched to the suggested version above, it is much clearer than the original version. 
- *Conclusions, line 6: 'an' informative precursor*
- **Response:** Fixed, thanks- good catch.
